# Study Protocol on Cognitive Performance in Bulgaria, Croatia, and the Netherlands: The Normacog Brief Battery

**DOI:** 10.3389/fpsyg.2016.01658

**Published:** 2016-10-27

**Authors:** Lea Jakob, Lana Bojanić, Desislava D. Tsvetanova, Eike K. Buabang, Nienke J. de Bles, Alexandra Sarafoglou, Annet Dijkzeul, Rocio Del Pino

**Affiliations:** ^1^Department of Psychology, Centre for Croatian Studies, University of ZagrebZagreb, Croatia; ^2^Department of Psychology, Faculty of Humanities and Social Sciences, University of ZagrebZagreb, Croatia; ^3^Department of Psychology, Sofia University “St. Kliment Ohridski”Sofia, Bulgaria; ^4^Institute of Psychology, Leiden UniversityLeiden, Netherlands; ^5^Department of Psychology, University of AmsterdamAmsterdam, Netherlands; ^6^Department of Psychology, Utrecht UniversityUtrecht, Netherlands; ^7^Department of Methods and Experimental Psychology, University of DeustoBilbao, Spain

**Keywords:** brief battery, cognitive performance, cross-cultural, neuropsychological assessment, Normacog

## Abstract

The Normacog Brief Battery (NBB) provides a comprehensive overview of an individual’s cognitive functioning within a short amount of time. It was originally developed for the Spanish population in Spain. However, there is a considerable need for brief batteries in clinical neuropsychological assessment, especially in eastern European countries. Cultural background and other individual characteristics—such as age, level of education, and sex—are shown to influence both cognition and patients’ performance on neuropsychological tests. Therefore, it is important to develop understanding of how and why culture impacts on cognitive testing and determine which sociodemographic variables affect cognitive performance. The current study aims to translate, adapt, and standardize the NBB in Bulgaria, Croatia, and the Netherlands, and to analyze the effect of sex, age, and education level on cognitive performance between these three countries. This brief battery assesses eleven cognitive domains, including those most currently relevant in cognition such as premorbid intelligence, attention, executive function, processing speed, and memory. The translation and adaptation of the battery for different cultures will be done using the back-translation process. After exclusion criteria, the current study will include a total sample of 300 participants (≥18 years old). The samples of 100 participants per country will be balanced through the consideration of their age and level of education. Effects of the sociodemographic variables (age, level of education, and sex) on cognitive performance are expected. Furthermore, this relationship is expected to differ across countries. A multivariate hierarchical linear regression will be used and exploratory analysis will be carried out to investigate further effects. The results will be particularly valuable for future research and assessment in cognitive performance. The growing demand for accurate and fast neuropsychological assessment shows the importance of creating a universal brief assessment tool for wider cross-cultural application.

## Introduction

Neuropsychological assessment is a performance-based method that is used to obtain information about a person’s cognitive functioning in different domains, such as memory, attention, processing speed, executive functions, spatial, and language functions (Harvey, 2012). A neuropsychological brief battery is a set of cognitive tests that provides a more complete profile of such cognitive functions. These batteries are used for several different purposes, including the acquisition of differential diagnostic information, assessment of treatment response, prediction of functional potential, and functional recovery (Harvey, 2012; Lezak et al., 2012). Therefore, it is of great importance that a person’s assessment is conducted and interpreted correctly. In order to correctly interpret the outcome of a cognitive test, a participant’s score must be compared to scores of a similar group. However, the availability of standardized tests and measures as well as norms is very limited when different populations have to be assessed (Nell, 1999). Many neuropsychological tests and measures have been developed for Caucasian, well-educated people, native English-speakers, and middle to upper class citizens, and consequently do not have the same diagnostic accuracy when used within other populations (Manly, 2008). In order to supply the lack of standardized brief batteries, the Normacog Brief Battery-NBB was created as part of the Spanish Normacog project using 700 healthy participants for validation (Del Pino, 2014; Del Pino et al., 2015a). The NBB presented high internal consistency in providing standardized norms for cognitive performance for Spanish speakers in Spain. These norms were adapted according to the sociodemographic characteristics of the country, and the results of the study showed a significant effect of age and level of education on the cognitive performance with no significant effect of sex. Furthermore, it is known that cultural background and related factors influence cognition and outcome of neuropsychological tests (Nell, 1999; Pérez-Arce, 1999; Ardila, 2007). According to Nell (1999), Ardila (2005), and Del Pino et al. (2015b), it is important to use calibrated norms for neuropsychological measures in different linguistic and cultural groups, and to account for the effect of culture on cognitive testing in order to provide correct interpretation of results. Sociodemographic characteristics—such as age, level of education, and sex—have been shown to influence cognitive performance (Mann et al., 1990; Collins and Kimura, 1997; Park et al., 1999; Reilly, 2012; Ojeda et al., 2014). However, the influence of these variables in cognitive performance seems to vary across cultures.

Regarding the influence of age on cognitive performance, Park et al. (1999) argued that differences in cognitive performance across cultures might disappear with increasing age, because of an overall cognitive decline. However, Ojeda et al. (2014) found systematic differences in cognitive performance between elderly Spanish and American individuals. The authors suggested that historical experience of political oppression and cultural background (like the attitude to make as few errors as possible) may have influenced the variation. Only older cohorts showed these discrepancies (Ojeda et al., 2014).

Considering education level, it has been shown that people with higher education perform better on neuropsychological tests than those from lower educational groups. This effect is most prominent in verbal neuropsychological tests (Lezak et al., 2012). Although, performance on non-verbal neuropsychological tests is also influenced by educational level when individuals with different educational levels within the same cultural group are compared (Rosselli and Ardila, 2003). However, despite the fact that educational level correlates with performance on some neuropsychological tests, it is not systematically related to everyday problem solving, which is a functional criterion of intelligence (Cornelius and Caspi, 1987). According to Rosselli and Ardila (2003), individuals with different levels of education have developed different ways of learning. Education could thus be considered a type of subculture. The development of different types of skills is influenced by culture, which results in different learning styles (Ostrosky-Solis et al., 2004). Furthermore, there seems to be an interaction between age and education. Groups with lower level of education start showing cognitive decline earlier in life while the cognitive functioning of better-educated groups tend to show decline at later age (Joao et al., 2016). Specifically, education seems to have a protective function against cognitive decline for general mental status but no for more complex tasks that require verbal abilities or working memory (Alley et al., 2007). While more years of education might be associated with higher scores in verbal abilities, working memory, and processing speed, there are no long-term effects of education regarding cognitive decline in any of those domains (Zahodne et al., 2011). In general, there seems to be no moderating role of education on cognitive decline directly; instead people with a higher level of education show a delayed in their cognitive impairment due to a higher baseline performance (Wilson et al., 2009; Lenehan et al., 2015). Another possible explanation for this phenomenon is that groups with lower educational attainment tend to have less intellectually stimulating jobs and lifestyles, resulting in a more rapid decline in cognitive ability (Heaton et al., 2009).

Another variable connected with cognitive performance is the participant’s sex. However, sex differences vary in magnitude across countries (Kimura, 1999). For instance, mental rotation and line angle judgment performance were assessed in more than 90,000 women and 111,000 men from 53 nations: males from wealthier nations demonstrated greater spatial abilities (Lippa et al., 2009). Another study conducted by Weber et al. (2014) in Europe showed that the magnitude of sex differences varies systematically across birth cohorts and regions. These variations were associated with changes in living conditions and cognitive stimulation over time. Weber et al. (2014) suggested that females benefit more than males from these societal improvements because females start from a more disadvantaged level than males.

In addition, socioeconomic status has already been proven to influence cognitive performance (Bradley and Corwyn, 2002; Haan et al., 2011; Eryigit-Madzwamuse et al., 2014). According to the literature reviewed, its effects begin as early as the prenatal period and continue throughout life. The strongest association between socioeconomic status and neurocognitive performance is found for language (Noble et al., 2007). According to Noble et al. (2007), this association could be due to the fact that the brain regions involved in language processing have a longer maturation period *in vivo* than any other brain region, which makes them more susceptible to environmental factors that covary with socioeconomic status. Another influential factor is individual professional activity. The literature suggests that the longer the period an individual has not been professionally active, the greater their decline in cognitive functioning is (Adam et al., 2013).

Exactly how age, level of education, and sex may influence cognitive performance is still an open research question. The effects are either contradictory or not stable. However, the authors of the aforementioned studies do agree that differences in cognitive performance, if they occur, are due to exposure to different educational opportunities, living standards, and historical backgrounds (Ojeda et al., 2014). Therefore, there is consensus that variations in cultural environment drive the significance of variables such as age and sex. For instance, cultural differences have been identified between Eastern and Western European countries, primarily in terms of collectivism and individualism, respectively (Kolman et al., 2003; Lykes and Kemmelmeier, 2014). Studies have shown that countries from Eastern Europe, such as Bulgaria and Croatia, are more socially interdependent than those from Western Europe, and place less importance on values such as mastery and autonomy while countries from Western Europe, such as the Netherlands, are thought to be more independent (Kolman et al., 2003). Furthermore, research has linked cultural variation to differences in cognitive styles. It is suggested that social orientations leads to different patterns of cognition. Independent societies tend to be more analytic while interdependent societies are more holistic (Nisbett and Miyamoto, 2005). This indicates that Eastern Europeans have a more holistic cognitive style compared to Western Europeans, who are more analytical (Varnum et al., 2008). Considering the aforementioned results, this study aims to further analyze whether intercultural differences in cognitive functioning exist not only between the countries from Eastern and Western Europe but also within Eastern European countries. Therefore, comparison of the countries of Bulgaria, Croatia, and the Netherlands are considered as adequate for the purposes of this study.

This study will explore aforementioned characteristics to analyze the cross-cultural differences in cognitive performance in three different countries (Bulgaria, Croatia, and the Netherlands). However, considering the state of current scientific literature, it is relevant to analyze not only cultural differences between the countries investigated, but also the effects of sex, age, and level of education on cognitive performance. More precisely, it is of interest whether there is an interaction between sex and age and if this interaction is influenced by the level of education of the participants, when controlled for sociodemographic covariates.

Therefore, the goals of the study are specified as follows: Firstly, the current project aims to translate and adapt the NBB (Del Pino et al., 2015a) in Bulgaria, Croatia, and the Netherlands; Secondly, the study aims to test both cross-cultural differences and the interaction between sex, age, and level of education on cognitive performance.

## Materials and Equipment

### Measures

#### Structured Interview

A structured interview was designed to collect the sociodemographic characteristics from participants and to make an informed decision regarding inclusion and exclusion criteria. The Hollingshead four-factor index of socioeconomic status (SES) will be used to measure each participant’s SES based on four domains: marital status, retirement/employment status, educational attainment, and occupational prestige (Hollingshead, 1975, unpublished).

#### Normacog Brief Battery

Neuropsychological data will be obtained by administering the NBB (Del Pino et al., 2015a) to participants from Bulgaria, Croatia and the Netherlands that meet the inclusion criteria (see paragraph Participants). The battery assesses eleven cognitive domains using eight subtests, listed below. The process of translation and back-translation will be carried out for several subtests, since they were not available in specific languages. The eight subtests forming the NBB are as follows (**Table 1**).

**Table 1:** Tests included in the Normacog Brief Battery (Del Pino et al., 2015a).

Cognitive domain	Normacog Brief Battery
Prospective memory	Prospective Memory Test (Einstein and McDaniel, 1990)
Premorbid functioning	Word Accentuation Test (Gomar et al., 2011)
General cognitive status	Montreal Cognitive Assessment (Nasreddine et al., 2005)
Attention and interference resistance	University of Deusto Interference Test (Ojeda et al., 2013. Based on Stroop Test, Stroop, 1935; Golden, 2001)
Visuoconstructive abilities and visual memory	Taylor Complex Figure (Taylor, 1969)
Executive function and mental flexibility	Modified Wisconsin Card Sorting Test (Schretlen, 2010)
Processing and perceptual speed	Salthouse Perceptual Comparison Test (Salthouse, 1991)
Semantic fluency	Semantic fluency subtest of the CIFA Test (Schretlen and Vannorsdall, 2010)

– The Prospective Memory Test (PMT) (Einstein and McDaniel, 1990) aims to assess prospective memory. Participants are instructed to remember performing an intended action (asking the examiner to return their keys or other personal item) at a particular time in the future (at the end of the testing). The participants’ ability to recall the instruction is scored according to the level of help from the examiner (from 0: no help; to 4: the examiner asks some questions to help but the examinee does not remember the assigned task).
– The Word Accentuation Test (WAT) (González Montalvo, 1991; Del Ser et al., 1997; Gomar et al., 2011) is the Spanish adaptation of the National Adult Reading Test (NART) (Nelson and O’Connell, 1978; Nelson, 1982). It aims to assess premorbid cognitive functioning by correctly reading aloud 30 low frequency words whose graphic accents have been removed. The total score is the number of words correctly read (from 0 to 30). The Dutch Adult Reading Test (DART) (Schmand et al., 1992) is a Dutch-language version of the NART that is being used for the Dutch sample. This test aims to determine premorbid intelligence by asking the participant to correctly read aloud 50 words that do not follow regular pronunciation rules. The total score is the sum of words pronounced correctly and can range from 0 to 100, with two points awarded for every correct word. Note that for the Bulgarian and Croatian sample premorbid functioning will not be assessed, as no scales adapted for respective populations are available.
– The Montreal Cognitive Assessment (MoCA) (Nasreddine et al., 2005) is a 30-point brief screening tool for evaluating mild cognitive impairment. It aims to assess the general cognitive status of the participant in the following cognitive domains: attention and concentration, executive functions, memory, language, conceptual thinking, computing and orientation. The performance of the test takes between 5 and 7 min. A score of 26 points or more is considered normal.
– The Animal naming fluency subtest from the Calibrated Ideational Fluency Assessment (CIFA) (Schretlen and Vannorsdall, 2010), was used to measure semantic fluency. The subtest used in the NBB assesses semantic fluency through determining the number of animals that a participant can name in 1 min.
– The Taylor Complex Figure (TCF) (Taylor, 1969) was initially constructed as an alternate form of the Rey–Osterrieth Complex Figure test. It consists of two parts: first, the participant is asked to copy the TCF, which provides information on visuoconstructive abilities. The second part aims to evaluate visual memory by asking the participant to reproduce the figure on a blank sheet of paper with a 3-min delay. Both drawing tasks are timed and have time limits for completion: 4 min for copying the figure and 2 min for recall. The score in both tasks ranges from 0 to 36 points according to the accuracy of the drawn elements and their correct placement.
– The University of Deusto Interference Test (Ojeda et al., 2013), based on the Stroop Test, (Stroop, 1935; Golden, 2001) assesses attention and interference resistance. It consists of three parts, each lasting 30 s. Much like the Stroop Test, it requires participants to name colors and incongruently colored names of colors aloud to assess level of interference. This new version overcomes the limitations that the original version presents related to people with color blindness and reading difficulties in elderly people. This new version includes different colors to the original one (blue, black, and pink). In addition, this version is shorter (90 s in total). The time limit for each section is 30 s (15 s less than the original version). In order to improve the reading of elderly people, the stimulus in this new version are bigger than the original one, and there are less stimulus (64 instead of 100).
– The Salthouse Perceptual Comparison Test (SPCT) (Salthouse, 1991) assesses processing and perceptual speed. This is done with two 30-s trials where the participant’s task is to mark whether a pair of letter strings (three- or six-letter strings, respectively) is same or different.
– The Modified Wisconsin Card Sorting Test (M-WCST) (Schretlen, 2010) is a shorter, modified version of the Wisconsin Card Sorting Test (Grant and Berg, 1948) that assesses executive functioning and mental flexibility. This version differs from the standard one in many ways (e.g., the reduction in number of cards from the original 128-card deck to only 48 cards). This and other modifications reduce the risk of frustration and quitting among elderly and impaired individuals. The test requires participants to sort a deck of 48 cards according to one of several implicit rules while adapting the rule used depending on examiner feedback about whether the last card sorted was correct or incorrect.

## Stepwise Procedures

### Participants

Data will be collected from adults in three countries: Bulgaria, Croatia, and the Netherlands. The aim is to assess at least 300 participants in total, 100 from each country, after exclusion. Participants will be recruited by “word of mouth” from different geographical locations in each country. In addition, universities, companies, and retirement homes will be contacted to ensure a comprehensive sample of the population in each country.

The sample size will be chosen taking into account the suggested sample size by the epidemiologic program “EPI INFO” and it will be based on realistic time and location constraints that authors will face when obtaining participants. According to the size of population older than 18 years for each country (Bulgaria: 6,179,026; Croatia: 3,468,429; and the Netherlands: 13,563,456), the sample size will require at least 100 participants per country. The samples will be balanced considering two main demographic characteristics: age and education. Stratification will be done according to eight levels of age (18–25, 26–35, 36–45, 46–55, 56–65, 66–75, 76–80, >80 years old) and four levels of education (0–6, 7–10, 11–12, and >12 years) (Ivnik et al., 1997; Peña-Casanova et al., 2009; Del Pino et al., 2015b). The age ranges will be chosen considering the dynamics of cognitive performance throughout the lifetime. This demographic characteristic is also crucial for the purposes of this study, which is why the researchers will aim to collect as diverse a sample as possible. Levels of formal education were chosen considering the differences in the education systems in the three countries. The inclusion criteria for participants in the study will be the following: (1) people of both sexes; (2) at least 18 years old; (3) sufficiently developed reading and writing skills; (4) voluntary participation; (5) signed informed consent. Exclusion criteria include: (1) having medical history of physical or mental illness that can interfere with cognitive functioning; (2) severe cognitive impairment; (3) having sensory impairment that cannot be corrected using aids; (4) being addicted to drugs or alcohol; (5) not being a native speaker of the language in which the assessment is being carried out; (6) and being functionally illiterate.

### Ethics

The study was approved by the Ethics Committee at the University of Deusto, Bilbao, Spain, which is the coordinator of the study. The study has also been approved by Sofia University “St. Kliment Ohridski,” Bulgaria, Faculty of Humanities and Social Sciences, and the Centre for Croatian Studies, University of Zagreb, Croatia. The ethical approval has been submitted for Utrecht University, Netherlands, to which the evaluators in this study are affiliated. All subjects will be volunteers and will provide written informed consent prior to their participation in the study, in accordance with the Declaration of Helsinki.

### Design and Procedure

The main goal of the study is to translate and adapt the NBB into Dutch, Bulgarian, and Croatian. For this purpose, copyright for all subtests included in the original battery was obtained. The battery has been translated and back-translated from English into each language by proficient individuals (who have studied English at least the bachelor level and are native speakers of language for which the test is being adapted). In the Netherlands, most of the subtests were already available. Once the instructions and answer sheets for each subtest have been translated and back-translated, an instruction manual will be developed in each of the three languages. The examiners for each country were trained individually in neuropsychological assessment. The first author of the NBB, Rocio Del Pino, executed this training. There will be five assessing examiners who have been trained in the NBB; two examiners from the Netherlands, two examiners from Croatia, and one examiner from Bulgaria. A detailed plan was developed for sample recruitment, which will be closely followed in order to make the sample comparable across countries (see **Table 2**). The sampling plan was developed following the proportions of the recruitment plan by Del Pino et al. (2015b).

**Table 2:** Plan for sample recruitment for the whole sample.

Age	Years of education				*n*
	0–6	7–10	11–12	>12	
18–25 years old	0 (0%)	6 (~16%)	15 (~40.5%)	16 (~43%)	37
26–35 years old	0 (0%)	6 (~16%)	19 (~24.5%)	12 (~32.5%)	37
36–45 years old	6 (~16%)	8 (~22%)	12 (~32.5%)	11 (~30%)	37
46–55 years old	6 (~16%)	8 (~22%)	11 (~30%)	12 (~32.5%)	37
56–65 years old	8 (~21%)	9 (~23.5%)	9 (~23.5%)	12 (~31.5%)	38
66–75 years old	17 (~45%)	12 (~31.5%)	3 (~8%)	6 (~16%)	38
76–80 years old	20 (~52.5%)	12 (~31.5%)	3 (~8%)	3 (~8%)	38
>80 years old	18 (~47%)	14 (~37%)	3 (~8%)	3 (~8%)	38
	75	75	75	75	*N = 300*

*The proportions are relative to the previous sampling recruitment used for the Normacog Brief Battery (Del Pino et al., 2015b, p. 62).*

(1) The population older than 18 years old of each country (Bulgaria, Croatia, and the Netherlands) was reviewed in their corresponding National Institute of Statistics (Bulgarian National Institute of Statistics, Croatian Bureau of Statistics, and Centraal Bureau Statistiek the Netherlands).
(2) The sample size was calculated with “EPI INFO” program for a total sample of 23,210,911. According to this, at least 270 participants should be included in the study for a confidence level of 90% (Barlett et al., 2001).

$$n=\frac{Z^{2}*N*p*q}{N*d^{2}+Z^{2}*p*q}$$

  *n* = sample size
  *Z* = *Z*-score value for the selected confidence level
  *N* = Population
  *p* = probability of success
  *q* = probability of failure
  *d* = acceptable margin error
(3) Adhering to previous studies (Ivnik et al., 1997; Peña-Casanova et al., 2009; Del Pino et al., 2015b), the sample will be stratified according to eight levels of age and four levels of education.
(4) The final sample will consist of 100 participants per country; that is the adjusted sample after excluding participants that fail to meet the inclusion criteria.

**FIGURE 1:**
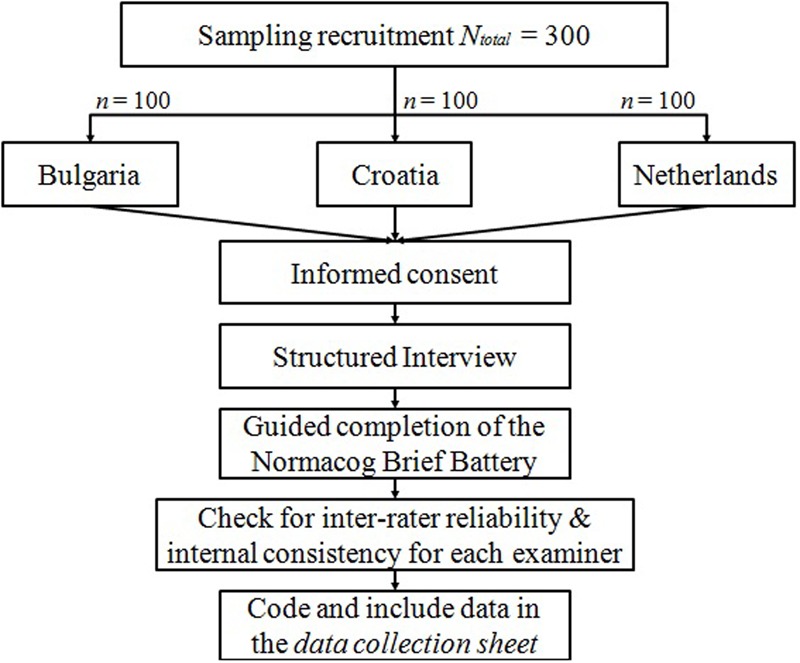
**Research design flowchart**.

**FIGURE 2:**
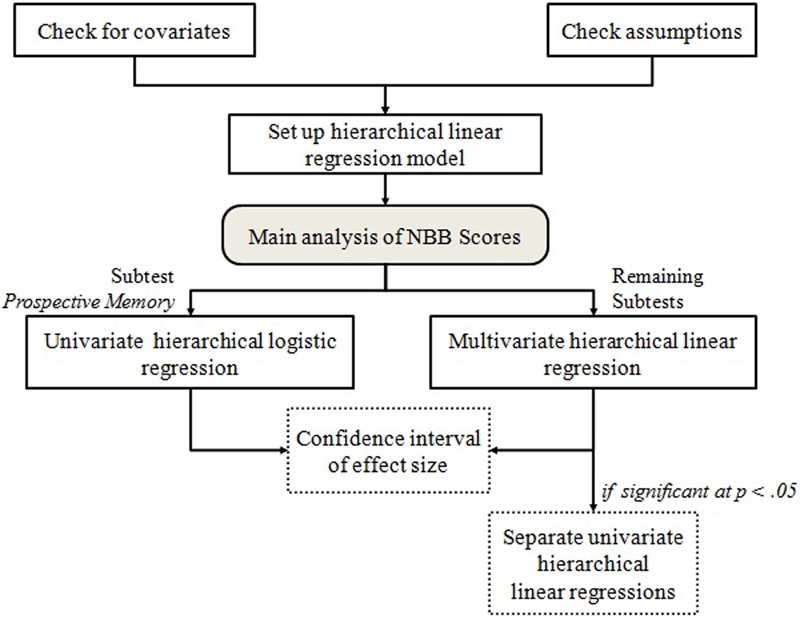
**Schematic illustration of the proposed analysis.** *Post hoc* analyses are displayed in dotted boxes.

The examiners will assess all participants in a quiet, neutral environment with minimum distractions (e.g., outdoor noises). The assessment itself will consist of two parts: a structured interview and the guided completion of the NBB (Del Pino et al., 2015a). A schematic illustration of the testing procedure is displayed in **Figure 1**. At the beginning of the study, all participants will have to read and sign the informed consent. They will then be interviewed by the examiner in order to gather demographic data and data related to their medical history, use of drugs, alcohol, etc. Once the interview is completed, the examiner will present all of the tests from the battery. The whole assessment procedure should last about 20 min with the majority of the participants, but with consideration of the fact that older participants are more likely take longer in completing the full battery. Neither the names nor any other information that may lead to the identification of the participants collaborating in the study will be published in any of the work resulting from this investigation. Therefore, each evaluator will have a code, as well as each participant. This code will be written in each sheet of the assessment in order to meet Data Protection Law and in accordance with the Declaration of Helsinki. To ensure that the coding scheme is comparable across examiners, the inter-rater reliability as well as the internal consistency will be checked. Finally, the complete data from the assessment will be codified and included in a data collection sheet. This data will then be processed and analyzed. A flowchart of the analysis plan is illustrated in **Figure 2**. All analyses will be performed using SPSS (version 19.0, 2010).

### Proposed Analysis

#### Controlling for Covariates

In order to identify possible covariates, it will be checked whether sociodemographic variables, such as SES, are equally distributed across countries. To do this, a Chi-squared test of homogeneity will be conducted for each of the sociodemographic variables. If significant deviations across countries are found, the corresponding sociodemographic variables will be included as covariates in the model.

#### Main Analysis

A hierarchical model will be used for the analysis of participant and country effects because the participants are hierarchically nested within countries (participants within each country perform more similar than participant between countries). Cultural differences as well as the interaction between sex, age, and level of education on cognitive performance will be tested with a hierarchical multivariate linear regression with multiple predictors. The proposed model includes the country of living as a predictor as well as the interaction between the predictors age, sex and education level. To control for their associated intraclass correlation country of living will be specified as a random factor. The predictors are expected to affect participants’ scores in the NBB. In addition, all sociodemographic variables that are unequally distributed across countries will be included as covariates in the model. A separate analysis will be carried out for the categorical variable resultant of the prospective memory test. A hierarchical logistic regression will be conducted to predict the probability that the participant needs help in the prospective memory task. For the logistic regression the same model is used as with the multivariate multiple regression. To validate the proposed model, a likelihood ratio test will be conducted to assess whether country of living and the proposed interaction contributes significantly to the model fit. The likelihood ratio test compares the model containing the proposed effect with the restricted model without the effect. This analysis will be executed both for the hierarchical multivariate regression as well as for the hierarchical logistic regression.

#### Proposed *Post hoc* Tests

Two *post hoc* tests are proposed for the main analysis. First, a *post hoc* power analysis will be conducted by analyzing the width of the confidence interval for the effect sizes. This analysis will indicate the likelihood of the real effect size being (non)zero. Second, the multivariate main analysis includes the standardized scores of all subscales in the NBB – except for the prospective memory – as an outcome variable. To obtain more detailed information about the location of the possible effect, separate univariate regressions will be conducted for each independent subscale score. The predictors will be the same as in the main analysis and corrections for multiple testing will be applied.

#### Additional Exploratory Analysis

In order to acknowledge the complexity of possible interactions, we will conduct additional exploratory analysis of the data, with the aim of gaining deeper insight into the interaction effects.

## Anticipated Results

Considering there is a serious lack of standardized neuropsychological instruments, we aim to ameliorate that situation by providing the interested parties with a readily translated and standardized brief battery. A review of currently available brief neuropsychological batteries has clearly demonstrated the demand for brief cognitive batteries in respective countries, especially in eastern European countries. A careful examination of the state of available neuropsychological instruments available for clinicians and other uses in Bulgaria and Croatia yielded no availability, while the Netherlands has limited availability of such resources. This will change once this battery is made available for use in the respective countries, providing professionals with a much-needed instrument.

With the growing understanding of the importance of accurate and fast neuropsychological assessment, the making of a universal brief assessment tool for a wider cross-cultural application will be put to test. We expect to demonstrate that cultural aspects—such as language, education, and age—affect individual cognitive performance and each country should take into account its own characteristics to provide accurate interpretation guidelines crucial for making appropriate clinical decisions. We expect our project to have impact across Europe, making the issue of assessment instrument availability acknowledged by professionals, encouraging parties involved in such assessment to tackle the issue by joining the Normacog development initiative in respective countries that will be supported from the original authors. This Protocol should serve as a starting point for any researcher who is interested in adaptation for their language.

Besides these strengths, some limitations have emerged so far. Obtaining an adapted test for premorbid functioning in both Bulgaria and Croatia has already proven to be problematic, which may complicate the comparability of results within the given domain. Further, the test for premorbid functioning used in the Netherlands consists of 50 words, while the same test used in Spain consists of 30 words, which might cause difficulties comparing the results. Secondly, another test, the Salthouse Perceptual Comparison Test, where a participant has to compare a series of letters with each other, may also be problematic in our study. This test has been made with the Latin alphabet while people in Bulgaria use the Cyrillic alphabet. For this reason, Latin letters were replaced with Cyrillic ones, potentially impeding comparison between people in Europe who did this test. Thirdly, the current study involves the minimum adequate sample size required for analyzing cross-cultural differences; therefore, if future studies aim to test a representative sample of each country, a larger sample size should be included.

Previous research suggests that cultural differences exist in cognitive functioning between countries from Eastern and Western Europe (Varnum et al., 2008). Therefore, the authors anticipate that such differences will also be found in the current research project. Considering the historical background of Eastern European countries, combined with better educational systems in the Western European countries, the authors expect that the overall cognitive performance in the Netherlands might be better than the one in Bulgaria and Croatia. However, the main goal of this study is not to measure and rank performance of these countries, but to determine if cultural differences between them actually exist. This finding would prove that it is necessary to create standardized culture-specific norms for each country in order to be able to validly interpret the results of the instrument.

To summarize, the translation, adaptation, and standardization of the NBB for the Netherlands, Croatia, and Bulgaria have been completed. Our next step is to gather participants and start the analysis of cross-cultural differences in cognitive performance.

## Author Contributions

All authors contributed equally to and have approved the final manuscript. RDP originated the study design as part of the Normacog project and was the supervisor of the study. LJ, LB, DT, NdB, and AD were responsible for collection of data. AS was responsible for the proposed analysis EB was responsible for writing the introduction and leading the copyright process. LJ was the team’s communication officer.

## Conflict of Interest Statement

The authors declare that the research was conducted in the absence of any commercial or financial relationships that could be construed as a potential conflict of interest.
